# Effectiveness of Chronic Wound Debridement with the Use of Larvae of *Lucilia Sericata*

**DOI:** 10.3390/jcm8111845

**Published:** 2019-11-02

**Authors:** Dariusz Bazaliński, Maria Kózka, Magdalena Karnas, Paweł Więch

**Affiliations:** 1 Father B. Markiewicz Podkarpackie Specialist Oncology Centre, Specialist Hospital in Brzozów, 36-200 Brzozów, Poland; darek.bazalinski@wp.pl; 2 Institute of Health Sciences, College of Medical Sciences of the University of Rzeszów, University of Rzeszów, 35-959 Rzeszów, Poland; 3 Department of Clinical Nursing, Faculty of Health Sciences, Collegium Medicum, Jagiellonian University, 31-501 Krakow, Poland; makozka@cm-uj.krakow.pl; 4 New Medical Technologies, Holy Family Specialist Hospital, 36-060 Rudna Mała, Poland; magdalena.karnas12@gmail.com

**Keywords:** chronic wounds, *Lucilia sericata*, *Phaenicia sericata*, wound debridement

## Abstract

The process of successful wound healing depends on effective debridement and infection control. One method of wound debridement, known since antiquity, is based on the use of fly larvae. Solid scientific evidence proves that maggot debridement therapy (MDT), like surgical intervention, can be effectively and safely used to remove necrotic tissue. Based on a review of the related literature, this study was designed to assess the effectiveness of chronic wound cleansing with the use of larvae of *Lucilia sericata (Phaenicia sericata).* Maggot therapy, applied in wound debridement and treatment, is a safe and effective method. Its benefits are associated with debridement, disinfection and faster tissue growth. MDT may reduce the duration of antibiotic therapy and the need for hospitalization, or it may decrease the number of outpatient visits required. It is a relatively cost-effective method, and, in addition to financial gains, it may reduce the frequency of inpatient treatment. In the literature, an increasing amount of scientific evidence confirms that such treatment can effectively reduce the biofilm and bacterial load in a wound.

## 1. Introduction

Without medical engineering, biotechnology, and pharmacy, developments in contemporary healthcare would not be as rapid and dynamic. Novel systems, drugs, and specialized instruments, constantly introduced into the medical sector, are designed to facilitate work, speed up the process of healing, and reduce the number of potential complications. Despite the efforts of experts focused on health and illness, available treatment options sometimes are limited to a minimum, or are quickly exhausted, particularly when dealing with an unknown or highly resistant microorganism. To address this, it is necessary to examine well known and widely used concepts, or look for novel experimental strategies. In recent years, people tend to associate fly larvae with something unpleasant, decaying carrion, or, perhaps, with barber surgeons or medicine men using unconventional healing methods. Treatment can only be administered using select species of flies. In accordance with current standards, the therapy uses larvae of the species *Lucilia sericata*, otherwise known as *Phaenicia sericata*. These represent a family of blow flies, feeding exclusively on necrotic tissues, which is a precondition of their medical application. Maggot debridement therapy (MDT) has not much improved in the past 80 years. It is true, however, that the therapy is effective, safe and affordable. Although, commercial producers, operating in countries where MDT is reimbursed by health insurers, seek to maximize their profits and offer maggots at prices approaching those of conventional advanced wound care dressings, it is still a highly effective, safe and affordable method of wound debridement, appreciated by patients and their families [1,2].

The use of maggots for medical purposes is not new. The oldest documents referring to interventions using larvae date from antiquity, and the first scholarly publications date from the 1500s, when the French surgeon Paré described the stages of healing of a large wound in the skull in the presence of maggots [3]. Subsequent comprehensive observations were mainly reported by military doctors; they described faster healing of wounds, with a lower risk of complications, in soldiers with open injuries colonized by fly larvae [4]. During World War I, mortality due to wounds and injuries amounted to nearly 70% among soldiers and civilians [5]. The antiseptic agents available at the time were insufficient. Both Baer and other military surgeons (Larey, Millingen, Keen, Zacharias) drew attention to wound debridement by maggots in wounded soldiers, but none of them conducted planned actions related to such a treatment concept [6]. After he was awarded the title of Professor of Orthopedic Surgery at John Hopkins University in 1929, Baer used his observations related to MDT acquired during the war [5]. He also showed that the therapy was effective in the treatment of chronic osteitis and osteomyelitis. He exposed the wounds of 21 patients to maggots and found that, 2 months after initiation of treatment, all of the patients’ wounds had healed well. To minimize the disgust of patients and personnel, and avoid migration of the larvae, he and his colleagues created net–cage bandages, covering the larvae. Additionally, special dressing was used to cover the border of the wounds, in order to decrease the strong itching effect caused by the maggots crawling around the wound [7]. The largest problems faced by the pioneers of this method were related to contamination of the larvae with *Clostridium tetani* and *Clostridium perfringens*. Baer identified the problem of microbial contamination and cross-infection in his patients and, to address this, he pioneered disinfection and aseptic rearing methods, to ensure high quality, sterile maggots for MDT. The low quality of the larvae and their potential contamination could have been one of many factors that, over time, resulted in a decline in interest in this method. Despite the positive effects of treatment, this form of therapy was not very attractive to patients and, especially, medical staff [6,8,9]. In the period 1930–1940, over 300 American hospitals introduced maggots into their wound management programs. At the same time, over 100 publications discussing MDT appeared. The discovery of penicillin by Fleming, and its rapid implementation by the pharmaceutical industry, as well as the implementation of innovative, minimally invasive surgical techniques, resulted in decreasing interest in MDT, which gradually disappeared between the mid-1940s and the 1980s, by which point it was rarely administered [10]. Larvae were used only in extremely difficult, frequently hopeless, clinical cases [11,12,13].

Only four years after the introduction of penicillin, over 50% of Staphylococcus aureus samples tested produced β-lactamase, making the bacteria resistant to most antibiotics. Increasing bacterial resistance has become a serious threat to civilization, resulting in a return to the search for more effective methods of destroying microorganisms. The observations and studies by Sherman and Pechter, regarding the elimination of bacterial flora, including MRSA (methicyllin-resistant *Staphylococcus aureus*), by larvae placed in the wound, opened new opportunities for researchers and clinicians worldwide [8].

William Baer was a pioneer in clinical larvae therapy, while Sherman, after over 40 years, has become the creator of a modern and safe concept of larvae therapy. In 1990, he opened a sterile laboratory at the Veteran Administration Hospital Medical Centre in Long Beach, California. His team carried out a prospective study, involving patients with pressure wounds following spinal cord injury, demonstrating that wound debridement with medicinal maggot larvae was more effective and required less time than conservative methods, whilst maintaining safety measures.^13^ Today, MDT is recognized as an effective and evidence-based method of wound debridement, stimulating wound healing [8,10,14].

In 2004, the Food and Drug Administration (FDA) listed larvae of *Lucilia sericata* as recommended agents for use in the treatment of chronic wounds, and, as such, they must satisfy the strict requirements specified for the pharmaceutical sector, including the relevant quality certificates [6,15]. Initially, recommended uses were limited to the process of wound debridement, since there was no clinical evidence related to effective disinfection and growth stimulation. Today, laboratory or clinical studies show antibacterial and/or growth stimulating applications of the therapy [16,17,18,19,20,21].

In Poland, MDT is still seen as an unconventional method; therefore, it is not subject to insurance refunds. The situation is further complicated by the fact that there are no standard treatment protocols or tools enabling subjective assessment of the method by patients. In our opinion, healthcare professionals also do not seem to be interested in using the method in practice.In fact, access to this type of treatment is largely limited, because MDT is offered in only a few places in the country. Conversely, in Germany and in the UK, the therapy is available within the national health care system, and the specialized dressings are produced by more laboratories. This means that the accessibility of this method is growing, while treatment costs are decreasing, as companies compete to attract patients. The growing demand for related products results in the decreased overall cost of treatment with the use of larvae, as well as the greater practicality of MDT compared to other methods, which can be observed in recent decades, in other countries as well as in Poland [5]. For example, 100 larvae purchased from a free-range colony are sufficient to debride a wound that is 10–50 cm^2^ in size, with 5–10 larvae used per 1 cm^2^, which accounts for a cost of 120–200 PLN (26–44 EUR), depending on the producer (Biolab® or Biomantis®) [22]. Considering the cost of the sample therapy with 100 loose larvae, it is cost-effective for the patient from both an economic and a medical perspective. The cost of a biogag is higher, but beneficial to eliminate the view of larvae for the patient, which may increase acceptance of this treatment method. This cost must be multiplied by the number of therapeutic cycles. This estimate does not include costs related to medical personnel, dressing materials, or specialist visits every 24 h, on average, as recommended. A study by Soares et al. recruited a total of 267 people with leg ulcers; 94 were treated with loose larvae, 86 were treated with larvae in a biobag, and 87 were treated with larvae in hydrogels [23]. The mean follow-up time was 171 days. The average total cost incurred by the participants assigned to each of the study arms was 1833 GBP (SD 1978 GBP) for loose larvae, 1696 GBP (SD 1948 GBP) for biobag larvae, and 1596 GBP (SD 1861 GBP) in the group where hydrogels were used [23]. The work of Wayman et al. estimated the expenses of 12 patients, covering medical care, dressings, and larvae, to be GBP 491.87. For comparison, in the same analysis, the total expenditure in the group of patients treated with hydrogels was GBP 1039.53. The median treatment cost in the larval group was GBP 78.64, compared with GBP 136.23 in the control group (*p* < 0.05) [24].

## 2. The Mechanism of Action

Only *Lucilia sericata* larvae from certified farms can be safely used in medicine. Complicated procedures of egg disinfection, using chloroamine, povidone-iodine and sodium hypochlorite, are intended to ensure safety during use of this product [25,26,27,28,29]. MDT is based on three mechanisms, observed when *Lucilia sericata* are introduced to the wound: the mechanical removal of necrotic tissue, bactericidal and bacteriostatic activity, and promotion of the healing process. Reports from the last decade point out that physical contact with the wound is a less important effect of the larval presence within the wound, and is probably a negative effect for the patient, who may feel a physical presence and wiggling in the wound. Chemicals secreted by larvae initiate the process of bacterial elimination and remodeling of the wound bed [25,30,31].

**Figure 1:**
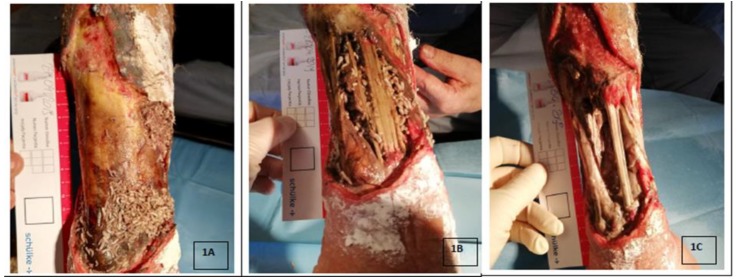
Process of debridement of a necrotic forearm wound with *Lucilia sericata* larvae; the wound has an area of nearly 200 cm², 100 free-range *Lucilia sericata* larvae from Biolab® culture were used. 1A. 24 hours since the larvae application, 1B. 48 hours since the larvae application, 1C. The condition after the removal of the larvae from the wound (over 72 hours). Note the exposed damaged ulna, and the extensor digitorum muscles. During therapy, pain sensations at the level of 2–3 Numeric Raring Scale (NRS).

Figure 1 shows the effect of debridement on a necrotic forearm wound measuring over 200 cm². Within 72 hours, 100 free-range larvae cleansed over 70% of necrosis in the wound, revealing the damaged forearm bones. The above observations suggest the potential use of this method in cases where wounds cover full skin-thickness, with a high risk of necrosis, or an already existing infection. The selection of an appropriate method for the further wound healing process is the next stage of local action [32].

A maggot does not literally consume pieces of tissue, but it secretes and excretes digestive enzymes (digestive secretions and excretions of arginase). Digestion begins directly in the wound bed, outside the maggot’s body. Dead tissue is liquefied and can be easily absorbed by the maggot [16]. This phenomenon can be easily observed during therapy because of the increase in the amount of exudate from the wound and its specific odor. Hobson was the first scientist to systematically present the proteolytic effects of digestive enzymes produced by the larvae of *Lucilia sericata* [33]. The movements of maggots, scratching tissue and secreting arginase, facilitate the debridement process. Other enzymes released include leucine aminopeptidase, collagenase and chymotrypsin-like proteases. These enzymes have various functions in the wound; they mainly transform dead tissue into liquid, which is either exuded from the wound or consumed by the maggots. The evacuation of necrosis and reduction in bacterial contamination promotes the regeneration process. The authors of numerous recently published papers have noted chemical substances that influence the promotion of the healing process. The migration of resident epidermal keratinocytes and dermal cells, including fibroblasts and dermal microvascular cells, from the wound margins into the wound bed, is a crucial step in wound healing. Amino acids, such as L-histidine, 3-guanidinopropionic acid and L-valinol, have been identified in larva secretions, and it has been demonstrated in vitro that these isolated components specifically increase the proliferation of human endothelial cells [34,35,36].

Collagenase speeds up the process of proteolysis by decomposing the material into smaller components, digested later by other enzymes secreted by the larvae [10,16]. Once necrosis is effectively removed, the consecutive stages of healing are facilitated by the increased phagocytic activity of leukocytes and higher oxygen pressure, resulting in optimum conditions for the regeneration of damaged tissues [37].

Proteolytic digestion is the initial act of tissue repair, leading to hemostasis, thrombosis, inflammatory cell activation and the reconstruction of tissue. Several studies suggest that the beneficial effect of larvae may be due to their ability to reduce pro-inflammatory factors [18,34,38]. The work of Pecivova et al. studied the effect of the *Lucilia sericata* salivary gland extract on opsonized neutrophils, stimulated with zymogen and unstimulated neutrophils. Salivary gland extract had no effect on superoxide production and myeloperoxidase release from unstimulated neutrophils, but significantly reduced both peroxide production and MPO secretion levels in zsonogen-stimulated opsonized neutrophils [39].

Following debridement, the wound may be appropriately assessed. Consequently, it is also possible to introduce other methods that promote wound closure, or to perform a skin transplant [37,40].

The granulation tissue in the wound, debrided and stimulated by larval secretions, should be dressed as soon as possible with the chosen method of local treatment. The choice of method depends on several factors, such as level of tissue destruction, area, wound etiopathogenesis, and general condition of the patient. Wound management criteria should be carefully implemented based on the TIME concept. The use of negative pressure wound therapy (NPWT) is effective in shrinking the wound, reducing exudate and bacteria, and stimulating neoangigenesis, specialized dressings, in the case of superficial and penetrative lesions that do not qualify for surgery [41,42,43]. There are clinical situations, especially in terminally ill patients, in which wounds debrided from necrotic tissue are only managed with dressings, due to the patient’s condition and poor prognosis [22].

The wriggling movement on the wound surface promotes neoangiogenesis and stimulates granulation. Larvae can move within a lesion due to their anatomy; they are covered with tiny spines and have mandibles in the form of small mouth hooks [44]. During therapy, most researchers report discomfort or mild pain in the range of 4% to 40% [1,2,37,40]. The larvae naturally inhabit decaying organic matter, such as dead bodies or excrement. Therefore, it is not surprising that maggots are well protected against the activity of various bacteria and toxins that occur in dead tissues. Greenberg suggested that antimicrobial compounds may be produced in a maggot’s gut by symbiotic microbes, such as *Proteus mirabilis*, and, in 1986, Erdmann and Khalil identified two antibacterial substances (phenylacetic acid and phenylacetaldehyde) in *Proteus mirabilis*, isolated from the gut of a related blow fly larva, *Cochliomyia hominivorax* [16]. By removing dead tissue, maggots speed up the healing process, as they consume and eliminate bacteria and, consequently, disinfect the wound. Disinfection is also enabled by other antibacterial properties, such as the capacity to produce bactericides (Lucifensin, Lucifensin II, Lucilin, Alfa-metoksyfenyl, MAMP) or agents that increase pH level (ammonia, calcium carbonate, ammonium carbonate), changing the pH in the wound. This results in deficiency of oxygen produced by bacterial flora, leading to the inhibition of bacterial growth in the more alkaline environment [25,44,45,46]. Feeding on bacteria, and the removal of bacteria, contributes to physical disinfection. However, larvae also produce bactericidal substances. Ammonia secretions lead to an increased pH, and the resulting alkaline environment promotes bacteriostatic effects. Antimicrobial activity was also observed in bacteria characterized by their high resistance to antibiotics, with the capacity to establish three-dimensional bacterial colonies (biofilm) [47,48,49,50]. Biofilm is an organized community of bacteria living closely in a protective polymer matrix. Biofilm-related infections are difficult to treat, even with advanced antibiotics [51]. Elimination of biofilms is important, because of their high resistance to penetration and the activity of the human immune system, as well as antibiotics. In chronic wounds, this is a particularly serious problem [45,52,53]. A bacterial colony is most effectively eliminated through its physical disruption and removal. Many experts recommend repeated debridement to remove biofilms (“*scraping*”), to get rid of the bacteria that occur in a wound [54,55,56,57,58]. This is also achieved by means of chemicals secreted by larvae foraging within the wound [16]. The work of Borkataki et al., in a study conducted experimentally in rats, observed that the infected wounds treated with MTD reduced significantly, with a decrease in bacterial load, compared to the control group of rats, treated with antibiotic only [17].

A study by van der Plas et al. showed that active components of maggot excretions/secretions (ES), at a very low concentration of 0.2 μg, disrupted the formation of a *Staphylococcus aureus* biofilm, and, at a higher concentration of 2 μg, the biofilms were degraded. Disintegration of the biofilm was observed at a concentration of 20 μg [49]. Research conducted by Chambers et al. has identified that the dominant proteolytic enzymes secreted by *Lucilia sericata* larvae are serine proteases (similar to trypsin and chymotrypsin) and metalloproteinase. In addition, the study showed that ES secretion can solubilize fibrin clots, as well as degrade laminin, type I and III collagen, and fibronectin. The authors concluded that ES proteolytic activity may promote wound healing through its remodeling effects on the wound, which promote the proliferation of fibroblasts [59].

A randomized controlled study using maggot therapy, conducted by Dumville et al., involving 267 patients with venous ulcers, did not show a significant difference in the bacterial load over time in the patients receiving maggot therapy, compared to the control group. Nevertheless, the treatment significantly reduced the total time needed for debridement, and the intensity of pain due to ulcers [60].

General recommendations for use of MDT include infected or necrotic wounds of varied etiologies, in cases where typical treatment, based on surgical necrectomy, is not feasible or advisable, or if overall benefits are expected to be poor. These conditions include pressure wounds with penetrating necrosis, venous ulcers, and Diabetic Foot Ulcers (DFU). Isolated case reports discuss the debridement of wounds associated with progressing cancer, as well as electrical burns [53,61,62,63,64]. By determining the time during which a larva feeds in the wound, where it is assumed that the larva imbibes 20–25 mg of tissue per day and feeds for three days, it is possible to estimate the initial quantity of dead tissue. Therefore, MDT may be used as an additional diagnostic method, to assess the quantity of necrotic tissue in a wound, and is more often used in forensics than in medicine [65].

MDT is not used in wounds without necrosis or with signs of granulation. These qualify for standard treatment, based on appropriately selected specialist dressings. Another contraindication is dry necrotic crust (eschar) with a hard and compact texture. *Lucilia sericata* cannot dissolve a solid surface. In this case, at the start of treatment, it is necessary to soften the crust, using compress with 0.9% NaCl, or specialist dressings (foam, hydrofiber infused with hydrogel), followed by surgical removal if demarcation is identified [20,60,66].

Strict analyses are conducted, and monitoring is performed, in patients with wounds located in the areas of the abdomen or the upper part of the digestive tract, as well as the eyes and the respiratory tract. Lesions situated in the vicinity of large arteries or deep, penetrating wounds, approaching internal organs, constitute a relative contraindication. In such cases, there is a greater risk of complications and/or failure of the therapy. 

Patients with DFU are a subject of interest for researchers and clinicians. The effectiveness and short time of wound debridement has been described in numerous studies [14,16,26,66,67,68].

According to Tian, MDT is an effective therapy for the treatment of DFU, but the scientific evidence is not strong enough to routinely recommend this form of local treatment [69]. Restrictions are also applied to patients diagnosed with coagulation disorders or those taking antithrombotic drugs. MDT is not administered to patients that are allergic to products used in maggot breeding (beer yeast, soy proteins) or during larvae disinfection [70].

## 3. Deposition of Larvae on the Wound

The start of MDT is preceded by targeted and thoughtful psychological preparation of the patient and his/her family. It is necessary to explain the purpose and recommendations of the selected therapy and to present its benefits and possible complications, as well as other sensations that may accompany the treatment [71,72,73]. Informed, written consent is of key importance when initiating the therapy. Acceptance of MDT is also assessed, using a questionnaire consisting of 10 questions, based on the Likert scale. This tool is part of the validation phase and is probably the first tool developed to assess the acceptance of this local treatment method [74]. Approximately 24 hours before the proper action starts, necrotic tissue should be softened by dressings dampened with 0.9% NaCl, and it is necessary to discontinue administration of any antiseptic agents [75].

**Figure 2:**
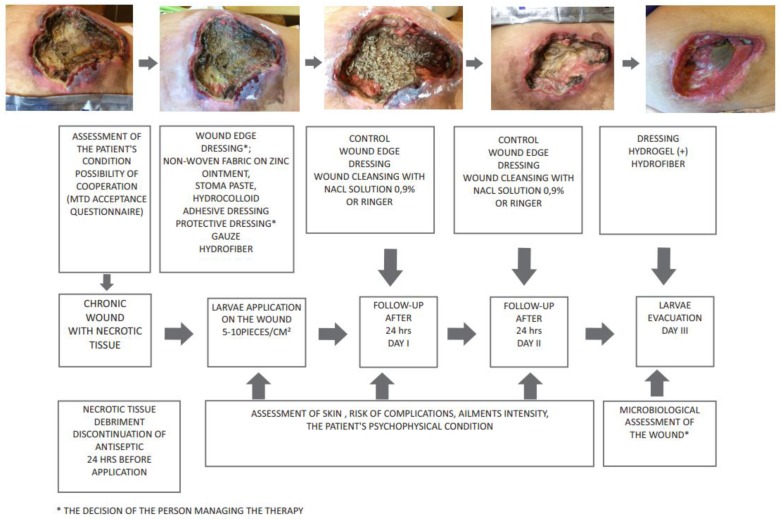
Algorithm of preparing and conducting therapy with *Lucilia sericata* larvae in a group [22].

At present, there are two methods of larvae deposition. A simple and less time-consuming method is based on the so-called “biobag,” i.e., a complete polyester bag with larvae [14]. There are also specialised dressings, with a hydrocolloid layer with a hinge structure. These reduce the risk that the larvae will escape from under the dressing, while enabling their complete penetration of the wound bed. These methods are also less stressful for patients [61,64,73]. Biobags and commercially available specialist dressings are not effective in penetrating wounds because larvae should have unobstructed access to the necrosis [26,76]. If the relevant area is particularly sensitive, or if the patient is afraid of larvae, they should only be kept in the wound for up to 24–48 h (this recommendation only applies to free-range larvae; the biobag should remain for 72 h). This leads to an increase in the cost and duration of the treatment, but the patient is not discouraged. Larvae can be introduced to the wound with two methods; free-range ones, using the syringe and cannula G14, 16, or directly onto the wound from the container (penetrating wounds, e.g., pressure sores, DFU). The second form of application uses a ready packaged biobag (5 × 5 cm, 10 × 10 cm) for direct application, usually on flat, non-penetrating wounds (e.g., leg ulcers, burns) [14,74]. Free-range larvae are most commonly used under so-called non-woven dressings (sandwich): free-range larvae skin protection (colloid applied as skin protection), sterile gauze dampened with 0.9% NaCl, sterile dry gauze, and bandages (preferably elastic). Instead of traditional gauze, the hydrofiber dressing moistened with 0.9% NaCl can be effectively used, which makes it easier to control the wound, compared to gauze, in which the larvae can migrate, especially during the debridement of superficial wounds [10,74]. Before the dressing is deposited, the skin around the wound should be secured with zinc ointment or alcohol-free stoma paste [10,26]. The protocol of larvae application and therapy is presented in Figure 2. 

If inadequately secured, the dressing may become loose, and the larvae may migrate and damage the skin around the wound. Irrespective of the method used, the larvae should not remain in the wound for more than 3–4 days [48,60,72]. Larval growth in the wound is not uniform and depends on nutrition; mature larvae leave the wound and migrate to, or out of, the dressing, which shows they must be evacuated from the wound. Pain sensations depend on the location and type of wound, as well as modifying factors [17,21,48,54]. Researchers emphasize the fact that patients may experience more severe pain than in conventional treatment [60].

An inspection should be performed at least every 24 h. Some authors do not provide information about inspection during the course of therapy. This does not mean that the dressing may be retained for 3–4 days, as there is a heavy amount of exudate. While checking the wound, it is necessary to assess the survival rate in the colony. The larvae are then irrigated from the wound using 0.9% NaCl solution, until optimum debridement is achieved [26,74]. Further treatment depends on the condition of the wound following biological debridement. General recommendations of reputable scientific societies should be observed, as well as the total potential cost of planned therapy and patient preferences [77,78,79,80].

## 4. Use of *Lucilia Sericata* Larvae in Wound Debridement

Some important meta-analyses discussing the effectiveness of MDT have been published in the past decade. One of these, carried out by Gray in 2008, included four studies. It was designed to determine the effectiveness of MDT in removing necrotic tissue, and its general impact on wound healing. All four studies reported that wound cleansing took less time if MDT was applied, compared to conventional treatments. The author reported that, due to the methodological limitations of these studies, the evidence is insufficient to allow a clear-cut conclusion that MDT is as effective, or more effective, than other debridement methods [81]. The work of Sun et al. performed a meta-analysis of 12 studies, based on PRISMA (Preferred Reporting Items for Systematic Reviews and Meta-Analyses) criteria. In each study, there were between 12 and 267 subjects, with a mean of 76 subjects. The studies were conducted in six countries (China, Great Britain, USA, Israel, Malaysia and Thailand) [26]. Some of the studies may have involved the same patients, yet their findings differed [60,82]. Out of 530 patients receiving MDT, 429 were subjected to another control therapy (hydrogel dressing). Eight studies qualified for the meta-analysis related to the healing rate with MDT. During the analysis of these studies, Sun et al. observed significant heterogeneity (*p* = 0.005, I_2_ = 65.5%). When compared to the control treatment, the pooled relative risk associated with wound healing when MDT was administered was 1.80 (confidence interval 95% 1.24–2.60). Subsequent analyses were focused on subgroups with various types of wounds. The pooled relative risk amounted was 1.79 (confidence interval 95% 0.95–3.38) in patients with DFU, and 1.70 (confidence interval 95% 1.28–2.27) in patients with pressure wounds and venous leg ulcers. The data collected from four studies showed that the time needed for healing was significantly shorter in the group receiving MDT than in the control group [60,66,83,84]. The pooled standardized mean difference in the healing time was 0.95 (confidence interval 95%, −1.24, −0.65, *p* = 0.502) [26].

Although previous studies encourage the use of MDT, there is still a shortage of credible evidence confirming its effectiveness [16,26,47,48,61,85]. The latest research on the cellular mechanisms involved in MDT (lucifensin, seraticin, chymotrypsin, DNAse, and unsaturated fatty acid), as well as genetic engineering techniques, could lead to the development of novel and innovative treatment methods. In the future, it is necessary to use this specific potential to design new wound management procedures [60,86,87,88]. Development of recombined and transgenic bioactive molecules from maggots is challenging, and an opportunity to increase approval for the treatment, and to improve results. It is likely that, in future, it will be possible to use specialized dressings composed of healing substances generated from larvae, without the need to apply maggots during treatment. The research of Linger et al., on the production and expression of human PDGF-BB protein, from two conditional expression systems in transgenic *Lucilia sericata* larvae, seems to be promising for future application. PDGF-BB protein was detectable in larval ES after induction. The results of the study can be used to deliver various growth factors and antimicrobial peptides to the wound environment to improve wound healing, and, according to the authors, thus improve patient treatment results in a cost-effective manner [89]. 

The development of biological therapy using *Lucilia sericata* larvae is gaining new supporters every year, among clinicians in various fields of medicine. Availability and unit price are improving. In our opinion, there is still a psychological and mental barrier associated with low interest, resulting also from the disgust of medical staff, especially women [74].

The world literature and researchers pay attention to the results of quantitative and qualitative research (evidence levels 1c and 3), and the patients surveyed accept larval therapy and even ask for such treatment [24,73,79,90,91]. Two qualitative studies have identified factors affecting acceptance, which include the time of occurrence or re-appearance of chronic wounds and their negative impact on quality of life; bad patient experience with other treatment methods; nurse–patient relationship; the experience of others; and being informed. The key factor that influenced refusal of treatment was the visual image of larvae, female sex, and senior age—over the age of 70 [73,92].

## 5. Conclusions

Larval therapy is a safe and effective method used for removing dead tissue during wound treatment. It is associated with debridement, disinfection, and faster tissue growth. MDT may reduce the duration of antibiotic therapy and the need for hospital admission, or it may decrease the number of outpatient visits required. This is a relatively cost-effective method, and, in addition to financial gains, it may result in other positive effects, such as a reduced number of beds occupied at a hospital ward. Recent scientific reports confirm biofilm and bacterial load reduction in the wound, as well as a positive effect on wound remodeling.

## Abbreviations

DFU	Diabetic Foot Ulcers
ES	Excretions/Secretions
EWMA	European Wound Management Association
MAMP	Alpha-methoxyphenol
MIC	Minimum Inhibitory Concentration
MDT	Maggot Debridement Therapy
MRSA	methicyllin-resistant *Staphylococcus aureus*
NRS	Numerical Rating Scale
NPWT	negative pressure wound therapy
PRISMA	Preferred Reporting Items for Systematic Reviews and Meta-Analyses
TIME	tissue, debridement, infection, moisture, edges
